# Effect of Bacterial Endotoxins on Superovulated Mouse Embryos In Vivo: Is CSF-1 Involved in Endotoxin-Induced Pregnancy Loss?

**DOI:** 10.1155/IDOG/2006/32050

**Published:** 2006-10-03

**Authors:** Yogesh Kumar Jaiswal, Madan Mohan Chaturvedi, Kaushik Deb

**Affiliations:** ^1^Molecular Biology and Reproductive Immunology Laboratory, School of Studies in Biochemistry, Jiwaji University, Gwalior, 474 011, India; ^2^Department of Zoology, University of Delhi, Delhi, 110 007, India

## Abstract

Mammalian embryonic development is regulated by several cytokines and growth factors from embryonic or maternal origins. Since CSF-1 plays important role in embryonic development and implantation, we investigated its role in gram-negative bacterial LPS-induced implantation failure. The effect of LPS on normal (nonsuperovulated) and superovulated in vivo-produced embryos was assessed by signs of morphological degeneration. A significantly similar number of morphologically degenerated embryos recovered from both nonsuperovulated and superovulated LPS treated animals on day 2.5 of pregnancy onwards were morphologically and developmentally abnormal as compared to their respective controls (*P* < .001. Normal CSF-1 expression level and pattern were also altered through the preimplantation period in the mouse embryos and uterine horns after LPS treatment. This deviation from the normal pattern and level of CSF-1 expression in the preimplantation embryos and uterine tissues suggest a role for CSF-1 in LPS-induced implantation failure.

## INTRODUCTION

A variety of cell types at the blastocyst implantation site produce growth factors that could play important role(s) in the implantation process. Decidual cells and/or embryos produce transforming growth factor alpha (TGF-alpha), epidermal growth factor (EGF), platelet-derived growth factor (PDGF), fibroblast growth factor (FGF), and colony stimulating factor (CSF-1) (Haimovici et al [1]). Furthermore, receptors for EGF, PDGF, and CSF-1 have been detected on embryonic and trophoblast cells. The receptor for CSF-1 (*c-fms*) has been detected from the early 2-cell stage embryo onwards albeit at low levels. CSF-1 mRNA transcripts have been detected in the oviducts and uterus suggesting a paracrine action of these growth factors during the preimplantation period of embryonic development (Arceci et al [2]).

During pregnancy, a paradoxical relationship exists between the fetal allograft and the maternal immune system. Several studies have demonstrated the adverse and beneficial effects of cytokines and growth factors secreted by activated lymphocytes and monocytes on the development of early embryos, during pathogenic conditions. These evidences have now formed the basis of the “immunotrophism” hypothesis, which suggests that local cytokines and growth factors produced by activated immune cells promote pregnancy. Colony stimulating factor-1 (or CSF-1, also known as macrophage colony stimulating factor, M-CSF) is found in humans as a 90 kd secreted homodimer and a 86 kd membrane-bound form, which is cleaved to release a 46 kd homodimer (Rettenmeir et al [3]). It has been shown that CSF-1 mRNA and its corresponding protein increase 1000 folds in the uterine horns during pregnancy in mouse (Pollard [4]). CSF-1 protein and CSF-1 mRNA expression also increase in the placenta in human with advancing gestation (Kauma et al 1999).

Adequate knowledge on preimplantation embryogenesis is necessary for the successful accomplishment of medically assisted reproductive technology and for embryo biotechnology (Iritani [5]). For carrying out the various embryonic manipulations *in vitro*, usually, a large number of viable and synchronously developing embryos are required and that can be obtained by superovulation. Silent subclinical gram-negative bacterial infections of the genital tract of pregnant animals may lead to poor quality of collected embryos. We designed the present study to evaluate the developmental status of preimplantation embryos developing *in vivo* in LPS-treated pregnant animals under nonsuperovulated/normal and superovulated conditions.

Gram-negative bacterial infections of the genitourinary tract of pregnant women are known to cause abortion, fetal loss, or poor pregnancy outcome (for a review see Deb et al [6]). Gram-negative bacterial endotoxins, lipopolysaccharides (LPS) is the main antigenic component of the gram-negative bacterial cell wall, and is known for its potency to activate the myeloid and nonmyeloid cells to produce cytokines. These cytokines and growth factors exert autocrine and paracrine effects on the surrounding cells and modulate the expression and synthesis of other cytokines. Silent subclinical infections of these bacteria can lead to early pregnancy losses where the mother remains unaware of it (see Deb et al [7]). In a previous study we have established the “minimum dose” of LPS which can compromise blastocyst implantation in mouse (Deb et al [8]). The objectives of this study were to find out if LPS could alter the pattern and level of mRNA expression of CSF-1 in the 2-cell to blastocyst stage embryos and uterine horns at narrow intervals over the preimplantation period of pregnancy in mouse.

## MATERIAL AND METHODS

### Superovulation and embryo recovery

Park strain mice were maintained, superovulated, and mated as described earlier (Deb et al [9, 10]). Female mice were killed by cervical dislocation on days 1.5, 2.5, 3.5, 4.0, 4.125, 4.25, 4.3, and 4.42 of the preimplantation period of pregnancy. The embryos were recovered on each day of pregnancy by flushing the excised oviducts and uterine horns with sterile PBS in sterile endotoxin free petri-dishes (Deb et al [10]). The recovered embryos were examined under a microscope (Nikon, Japan) and their morphology was studied using 20X and 40X objectives.

### Effect of the “minimum effective dose” of LPS on development of preimplantation stage embryos collected from nonsuperovulated/normal and superovulated pregnant animals

The “minimum effective dose” of LPS was given through IP route to normal and superovulated pregnant females on day 0.5 of pregnancy. The control animals were treated with equal volume of normal saline. The animals were sacrificed by cervical dislocation on days 1.5, 2.5, 3.5, and 4.375 of pregnancy and preimplantation stage embryos were recovered separately by flushing oviducts/uterus collected from control and LPS-treated animals of both the groups with PBS in endotoxin free petri-dishes. The embryos recovered from LPS-treated animals of both the groups during different stages of preimplantation period of pregnancy were counted and examined under a microscope (Nikon, Japan) for visible morphological abnormalities and compared with that of the respective control.

### Effect of LPS on expression of CSF-1 in embryos and uterus collected at different stages of preimplantation period of pregnancy

The experiments were performed to study the effect of LPS on the expression of CSF-1 in embryos and uterus collected at different stages of preimplantation period (ie, days 1.5, 2.5, 3.5, 4.0, 4.125, 4.25, 4.33, and 4.42) of pregnancy by RT-PCR. Park strain mice (6-7 weeks) were superovulated with PMSG and hCG as per the protocol. A “minimum dose” of LPS (ie, 5 *μ*g/animal) in 100 *μ*L of sterile normal saline (determined in a previous study) was injected to each pregnant animal through IP route on day 0.5 of pregnancy (Deb et al [8]). The control animals received 100 *μ*L of sterile normal saline in a similar manner. The animals of both groups were sacrificed at narrow intervals of the preimplantation period (ie, days-1.5, 2.5, 3.5, 4.0, 4.125, 4.25, 4.33, and 4.42) of pregnancy to collect the embryos and uterine horns. A total of about 500 embryos were collected at each time to detect the positive mRNA signals for CSF-1 by RT-PCR.

### Extraction of total RNA from uterus and embryos and semiquantitative RT-PCR

Total RNA was extracted from the embryos and uterine horns as described earlier (Deb et al [8]). The reverse transcription polymerase chain reaction (RT-PCR) was carried out using “Titan One tube RT-PCR System” (M/S Boehringer Mannheim, Germany). It was carried out as per the instructions provided by the manufacturer. The upstream and downstream primers for CSF-1 were initially purchased from M/S Clontech, USA, and later synthesized from M/S Genset Singapore Biotech Pte Ltd, Singapore. The primers for *β*-actin were synthesized from M/S Genset Singapore Biotech Pte Ltd, Singapore, and were used as internal control throughout the experiments (Weihua et al [11]). The primers used were 5′-CGGGCATCATCCTAGTCTTGCTGACTGTT-3′ plus 5′-AAATAGTGGCAGTATGTGGGGGGGCATCCT-3′ and 5′-GGGCACAGTGTGGGTGAC-3′ plus 5′-CTGGCACCACACCTTCTAAC-3′ for *β*-actin.

### Statistical analyses

Statistical analyses were performed using one-way analysis of variance (ANOVA) with Duncan's multiple range test for comparison of the significance level (*P*) between control and treated values. A *P* < .05 value was considered as significant difference between the values compared.

## RESULTS

### Effect of the “minimum dose” of LPS on development of preimplantation stage embryos collected from nonsuperovulated/normal pregnant animals

The embryos were recovered from reproductive tracts of control and LPS-treated animals during different stages of preimplantation period (ie, from day 1.5 to 4.375) of pregnancy to assess the effect of LPS on preimplantation embryonic development.

#### Day 1.5 of pregnancy

**Figure 1:**
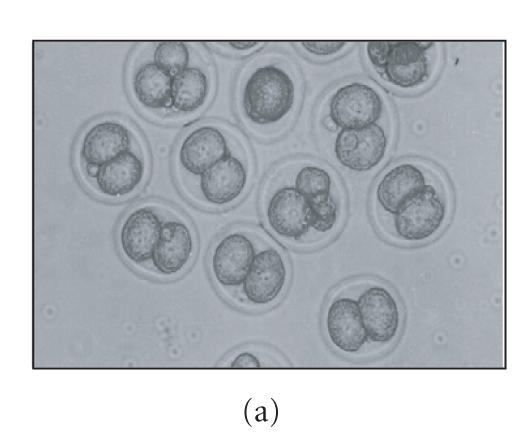

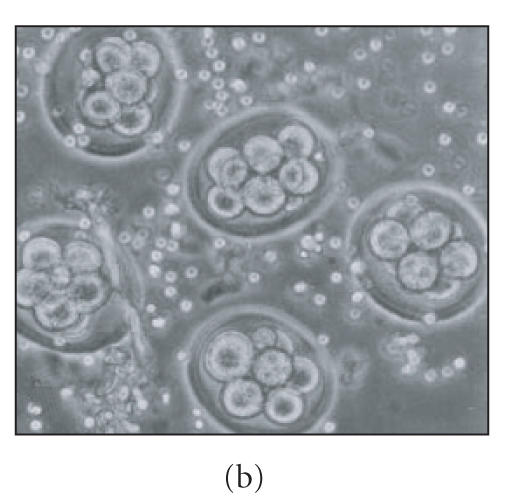

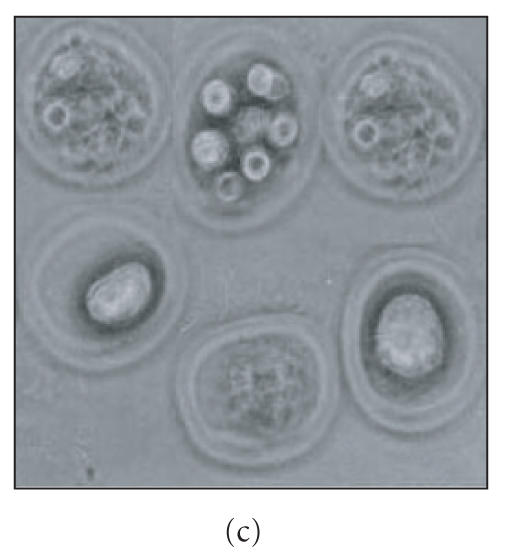

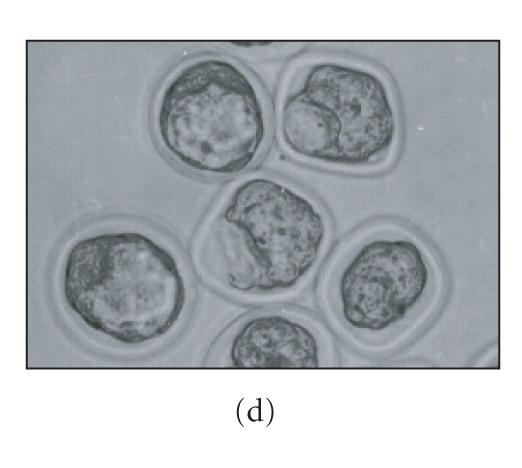

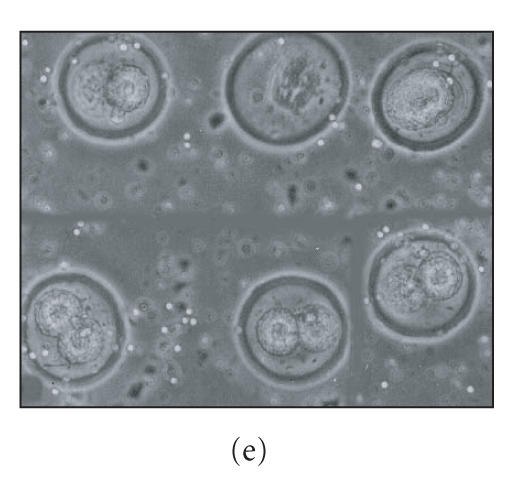

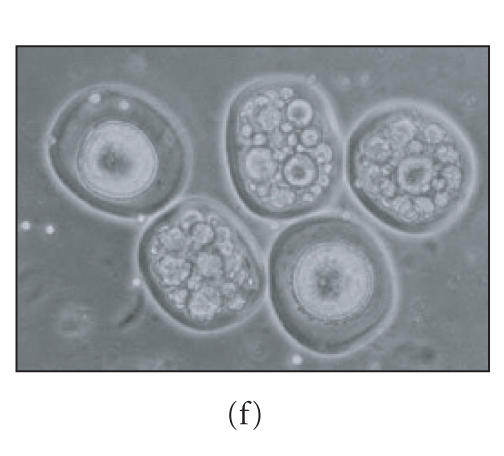

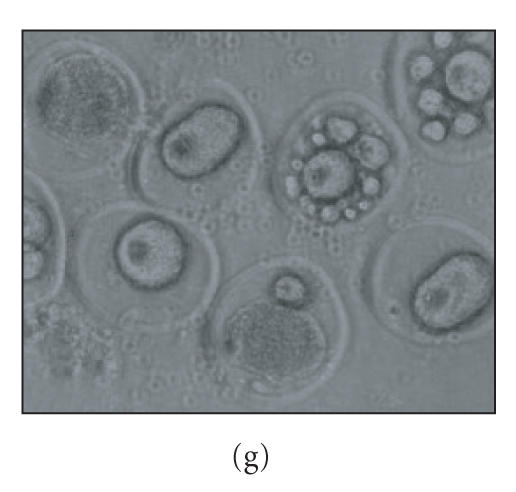

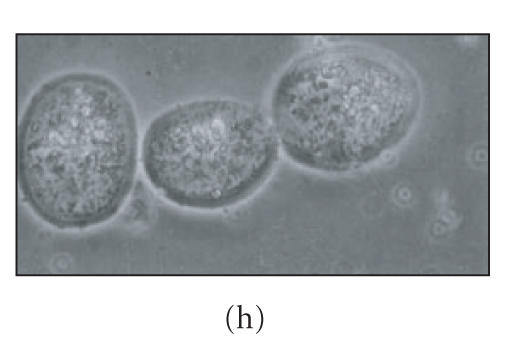
Photograph of embryos recovered from control and LPS-treated nonsuperovulated pregnant mice during different stages of preimplantation period of pregnancy. Panels (a), (b), (c), and (d) show embryos recovered from control animals on days 1.5, 2.5, 3.5, and 4.375 of pregnancy, respectively. Panels (e), (f), (g), and (h) show embryos recovered from the animals treated with the “minimum dose” of LPS on days 1.5, 2.5, 3.5, and 4.375 of pregnancy, respectively, X100.

Developmentally normal 2-cell stage embryos were recovered from oviducts of control and LPS-treated animals on day 1.5 of pregnancy (Figures 1(a), 1(e)). The average numbers of embryos collected from control and LPS-treated animals were found to be the same. Moreover, equal number of abnormal embryos was recovered from control and LPS-treated animals (Table 1).

#### Day 2.5 of pregnancy

The embryos recovered from oviducts of control animals on day 2.5 of pregnancy were normal and were at 4–8 cell stage of development (Figure 1(b)). However, developmentally arrested and apoptotic embryos with fully and/or partially degenerated blastomeres were recovered from oviducts of LPS-treated animals (Figure 1(f)). It was also observed that 4 ± 2.18% and 65 ± 5.022% of embryos collected from control and LPS-treated animals, respectively, were developmentally abnormal (Table 1). The average number of embryos retrieved from LPS-treated animals also declined by 15% as compared to that of the control (Table 1). A significant increase in the number of abnormal embryos recovered was observed from LPS-treated animals as compared to that of the control (*P* < .05).

#### Day 3.5 of pregnancy

The embryos were recovered from uterus instead of oviducts of control and LPS-treated animals on day 3.5 of pregnancy. The embryos recovered from control animals were at the morula stage of development. However, asynchronously cleaved and degenerated embryos with clear sign of apoptotic bodies were recovered from LPS-treated animals (Figures 1(c), 1(g)). It was observed that 7 ± 2.207% and 75 ± 8.66% of embryos retrieved from control and LPS-treated animals, respectively, were developmentally abnormal (Table 1). The number of embryos that was recovered on this day of pregnancy from LPS-treated animals was further reduced to 26% as compared to that of the control.

A significant increase in number of abnormal embryos recovered was observed from LPS-treated animals as compared to that of the control (*P* < .05, Table 1). However, no significant difference in collected abnormal embryos was found from animals treated with LPS on day 3.5 of pregnancy as compared to that on day 2.5 of pregnancy.

#### Day 4.375 of pregnancy

The embryos recovered from control animals on day 4.375 of pregnancy were at blastocyst stage of development with intact zona pellucida (ZP) and only 7 ± 2.207% of the collected embryos were developmentally abnormal (Figure 1(d)). However, 94 ± 5.56% of total embryos retrieved from LPS-treated animals were degenerated/fragmented and without ZP (Figure 1(h)). The average number of embryos recovered from LPS-treated animals was reduced as compared to that of the control. Therefore, 78% of embryonic loss was observed in LPS-treated animals as compared to that of the control on this day of pregnancy (Table 1). A significant increase in number of developmentally abnormal embryos recovered was observed from LPS-treated animals as compared to that of the control (*P* < .001). A significant increase in number of abnormal embryos recovered from LPS-treated animals was observed with increase in length of gestational period as compared to that of the control (*P* < .001, Table 1).

#### Effect of the “minimum dose” of LPS on development of preimplantation stage superovulated embryos collected from pregnant animals

The embryos were collected from reproductive tracts of superovulated control and LPS-treated pregnant animals during different stages of preimplantation period (ie, from day 1.5 to 4.375) of pregnancy to assess the effect of LPS on preimplantation embryonic development in superovulated pregnant animals.

#### Day 1.5 of pregnancy

**Figure 2:**
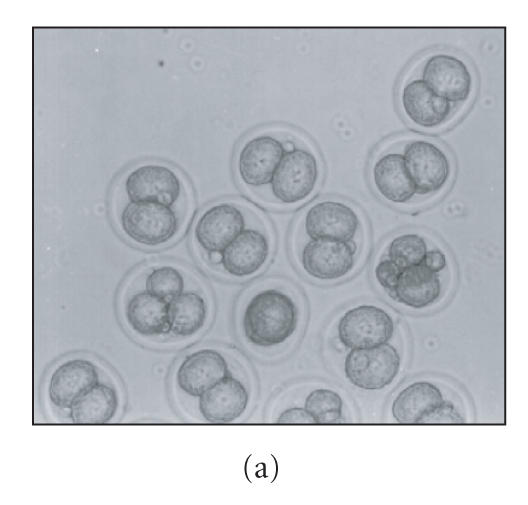

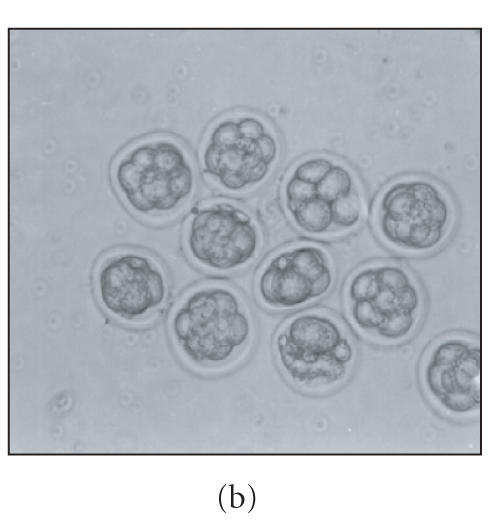

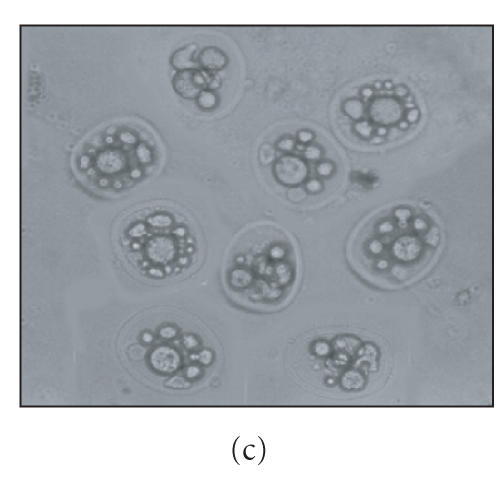

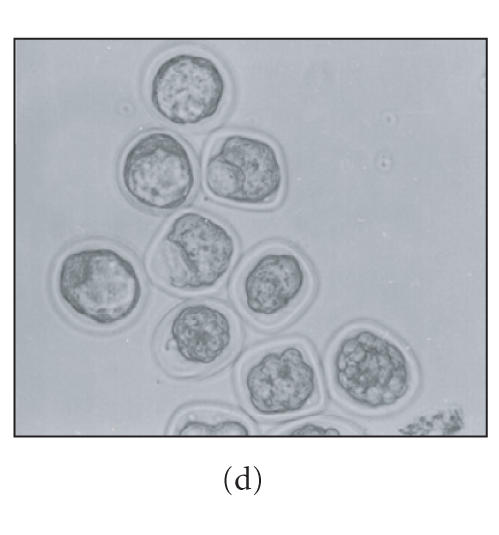

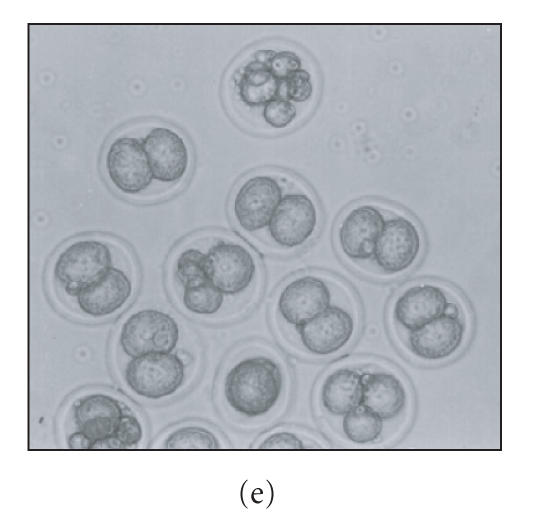

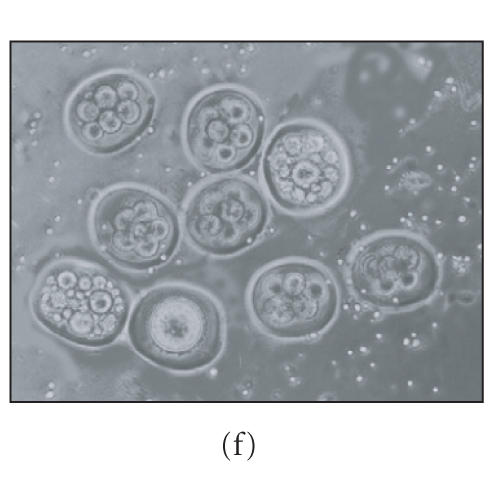

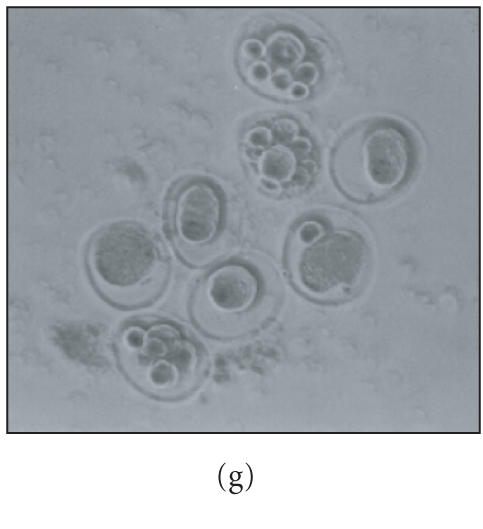

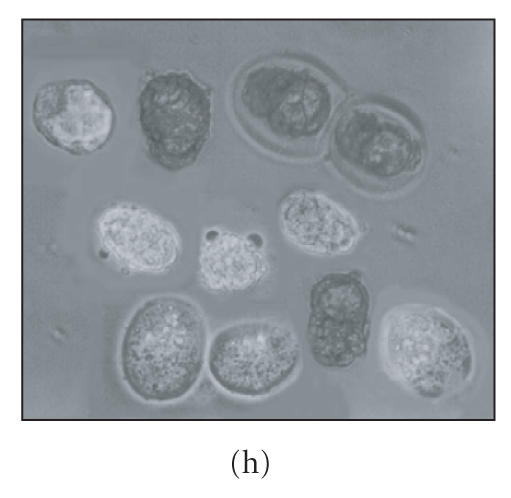
Photograph of embryos recovered from control and LPS-treated superovulated pregnant mice during different stages of preimplantation period of pregnancy. Panels (a), (b), (c), and (d) show embryos recovered from control animals on days 1.5, 2.5, 3.5, and 4.375 of pregnancy, respectively. Panels (e), (f), (g), and (h) show embryos recovered from the animals treated with the “minimum dose” of LPS on days 1.5, 2.5, 3.5, and 4.375 of pregnancy, respectively, X100.

An average of 29 ± 5 embryos was harvested from superovulated control and LPS-treated animals on day 1.5 of pregnancy. The recovered superovulated embryos from both groups of animals were normal and were at 2-cell stage of development (Figures 2(a), 2(e)). No significant differences in the quantity of developmentally abnormal embryos were observed in the total embryos recovered from control and LPS-treated pregnant animals on this day of pregnancy (Table 2).

#### Day 2.5 of pregnancy

The embryos recovered from oviducts of control animals on day 2.5 of pregnancy were normal and were at 4–8 cell stage of development (Figure 2(b)). However, developmentally arrested and apoptotic embryos with fully or partially degenerated blastomeres were recovered from oviducts of LPS-treated animals (Figure 2(f)). The average yield of superovulated embryos from control and LPS-treated animals was found to be similar on this day of pregnancy as compared to the previous day of pregnancy. However, a significant increase in the number of developmentally abnormal embryos recovered was observed from LPS-treated animals as compared to that of the control (*P* < .05, Figures 2(b), 2(f); Table 2). A significant increase in the number of developmentally abnormal embryos recovered from control and LPS-treated animals on this day of pregnancy was observed as compared to the embryos recovered from the previous day of pregnancy.

#### Day 3.5 of pregnancy

The embryos were recovered from the uterus instead of oviduct of control and LPS-treated animals on day 3.5 of pregnancy. The number of embryos harvested on this day of pregnancy from both groups of animals was similar as compared to the previous days of pregnancy. The normal embryos recovered from control animals were at morula stage of development. However, about half of the total embryos collected from LPS-treated animals were developmentally arrested, degenerated, and fragmented (Figures 2(e), 2(g)). A significant increase in the number of developmentally abnormal embryos recovered was observed from LPS-treated animals as compared to that of the control (*P* < .05, Table 2). However, the number of developmentally abnormal embryos from control and LPS-treated animals on this day of pregnancy did not differ significantly from the previous day of pregnancy.

#### Day 4.375 of pregnancy

The embryos recovered from control animals on day 4.375 of pregnancy were at blastocyst stage of development with intact zona pellucida (ZP) and only 8 ± 2.027 of the collected embryos were developmentally abnormal (Figures 2(d), 2(h)). However, the total number of recovered embryos declined by 10% as compared to that of the earlier days of pregnancy. The embryos recovered from LPS-treated animals were shrunken and without ZP. Moreover, the yield of embryos recovered was decreased by 38% as compared to that of the earlier days of pregnancy. It was found that 92 ± 5.566% of the total embryos recovered from LPS-treated animals were developmentally abnormal as compared to that of the control (Table 2). A significant increase in the number of developmentally abnormal embryos recovered was observed from LPS-treated animals as compared to that of the control (*P* < .05, Table 2). A significant increase in the number of recovered abnormal embryos from LPS-treated animals was observed on this day of pregnancy as compared to the previous day of pregnancy. Moreover, a significant increase in the number of abnormal embryos recovered from LPS-treated animals was observed with increase in length of gestation period as compared to that of the control (*P* < .001, Table 2).

### Expression of CSF-1 in preimplantation embryos and uterine horns

**Figure 3:**
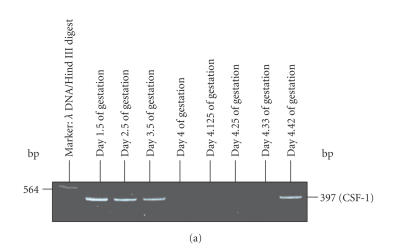

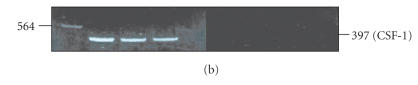

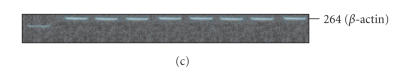
Detection of CSF-1 mRNA transcripts in the preimplantation mouse embryos collected from (a) LPS-treated animals and (b) control animals at the different stages of preimplantation period of pregnancy by RT-PCR. Pannel (c) shows a representative picture showing uniform expression of *β*-actin used as an internal control.

An average of 29 ± 5 embryos per pregnant animal was collected after superovulation. In the present study, about 500 embryos recovered during different stages of the preimplantation period (ie, days 1.5, 2.5, 3.5, 4.0, 4.125, 4.33, and 4.42) of pregnancy from control and LPS-treated animals were used each time to study the expression of CSF-1/M-CSF by RT-PCR. Positive mRNA signal of CSF-1 was observed in developing embryos collected from control and LPS-treated animals from day 1.5 to day 3.5 of gestation period (ie, from 2-cell stage to the morula stage) (Figures 3(a), 3(b)). However, an abrupt expression of this gene was again observed in embryos collected from LPS-treated animals on day 4.42 of pregnancy (Figure 3(a)). A uniform expression of *β*-actin gene was observed in the embryos collected from control and LPS-treated animals during different developmental stages of preimplantation period of pregnancy (Figure 3(c)).

**Figure 4:**
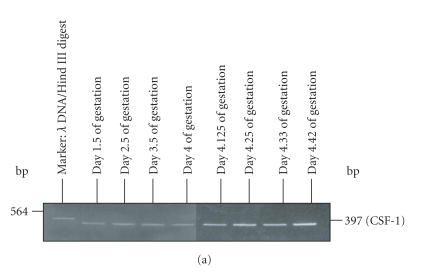

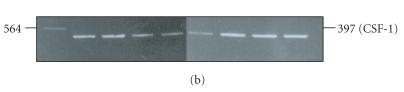

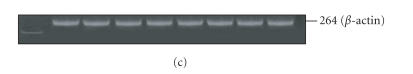
Detection of CSF-1 mRNA transcripts in the mouse uterine horns collected from (a) LPS-treated animals and (b) control animals at the different stages of preimplantation period of pregnancy by RT-PCR. Pannel (c) shows a representative picture showing uniform expression of *β*-actin used as an internal control.

The uterine horns collected from control and LPS-treated animals during different stages of the preimplantation period of pregnancy (ie, days 1.5, 2.5, 3.5, 4.0, 4.125, 4.33, and 4.42 of pregnancy) were used to study the expression of proinflammatory cytokines and growth factors CSF-1 by RT-PCR. Expression of CSF-1 mRNA increased gradually in uterine horns collected from control animals from day 1.5 of pregnancy till implantation (Figure 4(b)). In uterine horns from LPS-treated animals CSF-1 expression decreased between day 3.5 to 4.125 of pregnancy as compared to that of the control. However, its mRNA expression resumed back to normal levels (equivalent to control) from day 4.25 of pregnancy onwards till implantation (Figures 4(a), 4(b)). A uniform expression of *β*-actin gene was obtained in the uterus collected from control and LPS-treated animals during different developmental stages of pregnancy (Figure 4(c)).

## DISCUSSION

It has been shown that the pathophysiology of microbial infection that induced pregnancy loss in mouse is similar to that in human. Therefore, mouse may be used as a reliable and reproducible animal model to elucidate the molecular mechanisms of failure of pregnancy (Dudley et al [12]). To elucidate the mechanism of LPS-induced failure of implantation we investigated its effect on the *in vivo* development of preimplantation stage embryos collected from nonsuperovulated/normal and superovulated pregnant animals. We observed that the LPS had more or less similar effects on the quality and quantity of embryos collected from the nonsuperovulated and superovulated animals. The abrupt decline in the quality and quantity of the embryos in response to LPS on day 4.375 of pregnancy may be one of the causes of reduced reproductive efficiencies during gram-negative bacterial infections (Rupasri et al [13]).

The observed asynchronous cleavage and degeneration of preimplantation embryos recovered from LPS-treated animals may be due to activation of LPS-triggered apoptotic pathways in the cells of developing preimplantation embryos. Zou et al [14] suggested that LPS triggers F as mediated apoptotic pathway in mouse preimplantation (2-cell stage) embryos. It has also been reported that the expression of genes involved in cell death (eg, MA-3, p53, Bad, and Bcl-xS etc) gets elevated and that of the genes involved in cell survival (eg, Bcl-2) gets downregulated in embryos undergoing fragmentation (Jurisicova et al [15] and Levy [16]). The present study clearly demonstrates that LPS is associated with severe degeneration and fragmentation of mouse embryos *in vivo*. This may be due to an early expression of several proinflammatory cytokines in response to LPS in the developing mouse embryos.

The early preimplantation stage embryos are maintained in a nonadhesive state by a nonadhesive proteinaceous coat of ZP (Foulk [17]). The ZP prevents the attachment of the prematured embryo to the endometrium and also helps in the transfer of the developing embryos from the oviducts to the uterus (Carson et al [18]). The zona is shed from the surface of the matured blastocyst just before implantation (ie, on day 4.5 of pregnancy) under normal pregnancy and makes it attachment-competent. However, we saw an early loss of zona pellucida (on day 4.37 of pregnancy) from the developing embryos in response to LPS. The early loss of ZP may expose the premature blastocyst to high levels of cytotoxic factors, which may lead to the fragmentation and degeneration of developing embryos in mouse.

This study clearly implies that the low dose of LPS used in the present experiment may mimic the pathophysiology of subclinical genital tract infection of gram-negative bacteria, which may not affect the uterine preparation for embryonic receptivity at the gross anatomical or physiological levels. Our study also indicates that the preimplantation embryonic development is susceptible to low level of LPS *in vivo*. The present observations clearly demonstrate that LPS significantly affects the morphology and development of the superovulated embryos, which suggests the mouse embryos produced *in vivo* may be a good model to study such complications. It is possible that the growth factors and cytokines of embryonic and/or maternal origin, which are produced in response to LPS may alter the molecular dialogue at the feto-maternal interface, which may lead to poor pregnancy outcome and infertility in mouse.

CSF-1 performs several functions during implantation and placentation in physiological pregnancy. Bhatnagar et al [19], in an *in vitro* study, have shown that CSF-1 stimulates the development of trophectoderm of the blastocyst. CSF-1 regulates growth, differentiation, and functions of macrophages, is readily detectable in the peripheral blood in the steady state, and is further induced *in vivo* after infection (Cheers and Stanley [20]) or challenge with LPS (Roth et al [21]). Furthermore, CSF-1 treatment *in vivo* increased levels of LPS-induced TNF-*α* and IL-6 (Chapoval et al [22]).

We observed an early expression of CSF-1 gene in the embryos collected from the control animals. Our observation suggests its importance in the normal development of embryos during the early preimplantation stages. Zolti et al [23] reported the expression of CSF-1 in unfertilized human oocytes and during early embryogenesis. However, Arceci et al [2] could not detect its expression in preimplantation embryos in mouse. The pattern of CSF-1 expression in the embryos recovered from LPS-treated animals was similar to that of the control during days 1.5 to 3.5 of pregnancy. However, its abrupt expression in blastocysts (day 4.33) in response to LPS may lead to formation of developmentally compromised blastocysts which are incompetent for implantation.

The increase in CSF-1 production after ovulation and during pregnancy by uterine tissues as observed by us and previous researchers (Pollard et al [4]) may be due to increasing levels of progesterone, which is known to stimulate *in vitro* production of M-CSF in endometrium (Azuma et al [24]). The decreased level of CSF-1 mRNA in the uterus of animals treated with LPS as compared to that of the control animals on days 3.5, 4.0, and 4.125 of pregnancy indicates that LPS may have a slight antiprogestrogenic activity. The observed decrease in the expression of CSF-1, in the present study, in response to the treatment of animals with LPS may decrease uterine receptivity to the implanting blastocyst and may lead to unsuccessful implantation.

The ability of LPS to induce a differential regulation of the expression of CSF-1 in uterine horns and the preimplantation embryos suggests the possibility that the expressions of other cytokines like TNF and IL-6 were altered by CSF-1. Though the data is semiquantitative in nature, to our knowledge this is the first report which shows a differential expression of CSF-1 in the uterine horns and preimplantation embryos in response to LPS, and it highlights the importance of a regulated expression of CSF-1 for sustenance of normal pregnancy.

## Figures and Tables

**Table 1:** Effect of the “minimum effective dose” of LPS on development of preimplantation stage embryos collected from nonsuperovulated pregnant animals. *Data is expressed as mean ±* 1*SEM, with all values given in (%) of abnormal embryos*. Means bearing similar superscripted alphabets do not differ from each other at *P* ≤ .05 *(based on Duncan's multiple range test)*. *ANOVA*: treatment (T), F- value = 304.354 (*P* < .001), df 1,16; days of pregnancy (P), F-value = 42.761 (*P* < .001), df 3,16; T × P interaction F-value = 36.881 (*P* < .001), df 3,16.

Days of pregnancy	No of animals used	Control animals				LPS-treated animals			

		Total no of embryos recovered	No of abnormal embryos recovered	Loss in yield (%)	Abnormal embryos (%)	Total no of embryos recovered	No of abnormal embryos recovered	Loss in yield (%)	Abnormal embryos (%)

1.5	3	27 ± 2	0 ± 2	0	4 ± 2^(e)^	27 ± 2	0 ± 2	0	4 ± 2.17^(e)^
2.5	3	27 ± 2	0 ± 2	0	4 ± 2.18^(e)^	23 ± 2	15 ± 2	15	65 ± 5.022^(b)^
3.5	3	27 ± 2	2 ± 1	0	7 ± 2.027^(c)^	20 ± 2	15 ± 3	26	75 ± 8.66^(b)^
4.375	3	27 ± 2	2 ± 1	0	7 ± 2.027^(c)^	6 ± 3	6 ± 1	78	94 ± 5.56^(a)^

**Table 2:** Effect of the “minimum effective dose” of LPS on development of preimplantation stage embryos collected from superovulated pregnant animals. *Data is expressed as mean ±* 1*SEM, with all values given in (%) of abnormal embryos*. Means bearing similar superscripted alphabets do not differ from each other at *P* ≤ .05 *(based on Duncan's multiple range test).* *ANOVA*: treatment (T), F-value = 959.694 (*P* < .001), df 1, 24; days of pregnancy (P), F-value = 251.514 (*P* < .001), df 3,24; T × P interaction F-value = 195.104 (*P* < .001), df 3,24.

Days of pregnancy	No of animals used	Control animals				LPS-treated animals			

		Total no of embryos recovered	No of abnormal embryos recovered	Loss in yield (%)	Abnormal embryos (%)	Total no of embryos recovered	No of abnormal embryos recovered	Loss in yield (%)	Abnormal embryos (%)

1.5	4	116 ± 5	0 ± 5	0	2 ± 1.199^(e)^	116 ± 5	0 ± 6	0	3 ± 1.472^(e)^
2.5	4	116 ± 5	12 ± 5	0	9 ± 1.914^(d)^	116 ± 5	48 ± 5	0	42 ± 1.658^(b)^
3.5	4	116 ± 5	16 ± 5	0	13 ± 2.179^(d)^	116 ± 5	56 ± 6	0	48 ± 2.041^(b)^
4.375	4	104 ± 5	8 ± 4	10	8 ± 2.027^(c)^	72 ± 5	64 ± 4	38	92 ± 5.566^(a)^
